# Targeting mTOR and Src restricts hepatocellular carcinoma growth in a novel murine liver cancer model

**DOI:** 10.1371/journal.pone.0212860

**Published:** 2019-02-22

**Authors:** Sarah Walker, Miriam Wankell, Vikki Ho, Rose White, Nikita Deo, Carol Devine, Brittany Dewdney, Prithi Bhathal, Olivier Govaere, Tania Roskams, Liang Qiao, Jacob George, Lionel Hebbard

**Affiliations:** 1 Storr Liver Centre, Westmead Institute for Medical Research, Westmead Hospital and University of Sydney, Westmead, Australia; 2 Gastroenterology and Hepatology Unit, The Canberra Hospital, Woden, Australia; 3 Department of Molecular and Cell Biology, Centre for Molecular Therapeutics, James Cook University, Australian Institute of Tropical Health and Medicine, Townsville, Australia; 4 University of Melbourne, Victoria, Australia; 5 Translational Cell and Tissue Research, Department of Imaging and Pathology, KULeuven and University Hospitals Leuven, Leuven, Belgium; 6 Liver Research Group, Institute of Cellular Medicine, The Medical School, Newcastle University, Newcastle-upon-Tyne, United Kingdom; University of Navarra School of Medicine and Center for Applied Medical Research (CIMA), SPAIN

## Abstract

Liver cancer is a poor prognosis cancer with limited treatment options. To develop a new therapeutic approach, we derived HCC cells from a known model of murine hepatocellular carcinoma (HCC). We treated adiponectin (APN) knock-out mice with the carcinogen diethylnitrosamine, and the resulting tumors were 7-fold larger than wild-type controls. Tumors were disassociated from both genotypes and their growth characteristics evaluated. A52 cells from APN KO mice had the most robust growth *in vitro* and *in vivo*, and presented with pathology similar to the parental tumor. All primary tumors and cell lines exhibited activity of the mammalian target of Rapamycin (mTOR) and Src pathways. Subsequent combinatorial treatment, with the mTOR inhibitor Rapamycin and the Src inhibitor Dasatinib reduced A52 HCC growth 29-fold *in vivo*. Through protein and histological analyzes we observed activation of these pathways in human HCC, suggesting that targeting both mTOR and Src may be a novel approach for the treatment of HCC.

## Introduction

Liver cancer (hepatocellular cancer [HCC]), the fifth commonest in terms of cancer incidence and the second leading cause of cancer related deaths worldwide, is a poor prognosis cancer [1]. The major risk factors for HCC are hepatitis B virus (HBV) and hepatitis C virus (HCV) infection, and excessive alcohol intake that promote fibrosis and cirrhosis, and ultimately oncogenic transformation. However, in the last 3 decades, rising rates of obesity and the metabolic syndrome have led to dramatic increases in HCC related to non-alcoholic fatty liver disease (NAFLD) and its inflammatory form non alcoholic steatohepatitis (NASH). HCC arising in this context can develop in the absence of cirrhosis [2]. Over 1 billion people worldwide have NAFLD [3] and thus it is to be expected that NASH HCC will become the most common cause for liver transplantation and an increasing cause of morbidity [4]. The therapeutic options for all forms of HCC are limited with just 2 approved agents (Sorafenib and Regorafenib) that prolong life by a median of just 10–12 weeks [5–7]. Hence new therapeutic approaches based on an understanding of disease pathogenesis and molecular phenotype are urgently required.

A pathway that has been previously targeted is the mammalian target of Rapamycin (mTOR). mTOR is present in two different complexes mTORC1 and mTORC2; only mTORC1 is sensitive to Rapamycin. Active and phosphorylated mTOR phosphorylates 4E-binding protein (4E-BP1) and p70S6K translation initiation factors that are necessary for cap-dependent mRNA translation. This in turn increases the synthesis of numerous proteins required for cell cycle progression, angiogenesis, cell proliferation and growth. mTOR activity is also regulated by a number of oncoproteins and tumor suppressors, including AMP-activated protein kinase (AMPK), protein kinase B (AKT) and more recently, the non receptor tyrosine kinase (non-RTK) Src [8, 9]. Importantly, studies have shown that mTOR is upregulated in HCC [10, 11] and this has led to clinical trials of mTOR inhibitors or their combination with other drugs. However, the results have been uninspiring and offer no alternative to current therapeutic practices [12–14]. Considering that mTOR sits at the intersection of a number of signaling pathways and the diverse clinical and molecular heterogeneity of HCC, it is highly plausible that a myriad of feedback mechanisms, for example the sustained activation of AKT, can over-ride the specificity of a mTOR inhibitor. Thus, to treat HCC, a combinatorial approach may be required predicated on understanding the molecular phenotype of tumors as already shown for melanoma [15].

Another difficulty in the field is that the majority of HCC tumor models are cell lines derived from human HCCs. Although this a positive in representing the genetics and signaling pathways of human HCC, studies *in vivo* must then use immune compromised mice, that then leads to possible differences in responses from the host, and lacks the representative pathology of human disease. Thus, any advancement in available models could possibly lead to better therapeutic approaches. To address these issues concerning murine models and mTOR directed therapy, we chose to focus on the adipokine, adiponectin, whose serum levels are paradoxically reduced as body mass index (BMI) increases, and parallels dysregulated lipogenesis, liver fibrosis and hepatic inflammation and fatty liver disease [16, 17]. In genetic models, low serum APN can promote breast and colon cancer development through angiogenic mechanisms and by modulating the AMPK-activated protein kinase (AMPK)/mammalian target of Rapamycin (mTOR) pathway [18, 19]. In mice APN absence promotes liver tumor formation in mice fed a choline-deficient diet L-amino acid-defined diet or treated with the carcinogen azoxymethane [20, 21]. We treated APN null mice with diethylnitrosamine (DEN) and observed larger HCCs versus wild-type, and derived A52 cells from an APN KO tumor. Subsequent combinatorial inhibition of mTOR and Src with Rapamycin and Dasatinib dramatically reduced A52 tumor growth and importantly, we observed activity of the mTOR and Src pathways in human HCC. These data support the use of Dasatinib to synergize with the action of mTOR inhibitors to treat HCC.

## Materials and methods

### Animal studies

APN KO mice were sourced from the Matsuzawa lab [22] and bred onto the in house C57B/6 strain for 6 generations. HCC was induced at P15 with a single intraperitoneal injection of diethylnitrosamine 25 mg/kg (DEN; Sigma Aldrich). Mice were kept in a temperature-controlled facility with 12-hour light/dark cycles and were fed a standard rodent chow diet with water *ad libitium*. Animal coat texture and vital signs were examined three times per week and at experiment end euthanized by carbon dioxide inhalation 9 months after injection and examined for metastases. The livers were weighed and macroscopically assessed for tumor growth. Tumor incidence (>0.5mm) and total tumor volume were calculated (volume = width^2^ x length/2). Tumour cell injections were performed on mice anesthetized with ketamine (60mg/kg body weight) and xylazine (100mg/kg body weight). Animal experimental protocols were approved by the Western Sydney Area Health Service Animal Ethics Committee.

### Histology, immunohistochemistry, and image analysis

Murine tissue was fixed in 4% paraformaldehyde in PBS overnight, dehydrated, and embedded in paraffin. Sections were cut at 10 μm and stained with H&E and pathologic evaluation was performed independently by PB and TR. The other half was snap-frozen in liquid nitrogen and sectioned frozen at 7 μm for immunohistochemical analysis. Formalin-Fixed paraffin embedded human and mouse samples were stained using the Bond Polymer Refine Detection kit on the Bond Max autostainer (Leica) with antibodies against Ki67 (ab15580; abcam), p-mTOR (2976S; Cell Signaling) and p-4E-BP1 (236B4; Cell Signaling). Frozen sections were fixed in ice-cold acetone or 4% paraformaldehyde in PBS for 10 min and stained with phoshpo-histone H3 (ser^10^; 9701; Cell Signaling) and TUNEL (Roche), respectively and counterstained with DAPI. Human HCC tumors were sourced from the Westmead Liver Clinic and the Leuven Pathology tumor bank; non-cirrhotic liver tissue surrounding a colorectal metastasis was used as a control, and approved by the Westmead Human Ethics Committee and Ethical Committee UZ Leuven. H & E slides were scanned with a NanoZoomer 2.0-HT and images prepared in Photoshop. Immunofluoroescent images were taken using a Spot camera (Diagnostic Incorporated) on a Zeiss Axiovert 405M microscope and the density calculated with ImageJ software.

### RNA isolation, quantitative real time RT-PCR and serum analyses

Total cellular RNA was extracted from liver and tumor tissue, treated with DNase and resuspended in RNase free water, complementary DNA synthesized and real-time polymerase chain reaction carried out using specific primers for CD68; forward TGACCTGCTCTCTCTAAGGCTACA and reverse TCACGGTTGCAAGAGAAACATG; IL-6; forward TAGTCCTTCCTACCCCAATTT and reverse TTGGTCCTTAGCCACTCCTTC, and TNFα forward TCTTCTCATTCCTGCTTGTGG and reverse CACCCCGAAGTTCAGTAGACA and normalized against GAPDH, forward GTCGTGGATCTGACGTGCC and reverse TGCCTGCTTCACCACCTTC. The serum markers for glucagon, insulin, leptin, interleukin-6 (IL-6) and tumor necrosis factor-α (TNFα) were measured from the serum of WT and APN KO mice using the Luminex200 analyser according to the manufacturer’s instructions. Aspartate transaminase (AST) and alanine transaminase (ALT) were measured through the in-house pathology service.

### Protein extraction and western blotting

Frozen pairs of HBV and HCV human non-tumor and tumor, and 3 primary NASH HCCs were sourced from the Westmead Liver Clinic and approved by the Westmead Hospital Human Ethics Committee. Total protein extracts from murine and human tumor tissue were subjected to immunoblotting as previously described [23]. Antibodies for AMPK (#2532), phospho-AMPK (Thr172; #2535), Akt (#9272), p-Akt (Ser 473; #4051), mTOR (#2972), p-mTOR (Ser2448; #2971, 4E-BP1 (#9644), p-4E-BP1 (Thr70 and Ser65; #9455), p70S6-kinase (#9202), p-p70S6-kinase (Thr421/Ser424; #9204), Src (#2108), p-Src (Tyr416; #2101), AFP (#3903), JNK (#9252), p-JNK (Thr183/Tyr185; #9255) and PCNA (#2586) were from Cell Signaling. Anti-β actin (A2228) antibodies were from Sigma Aldrich, and anti-albumin (NB600-41532) was from Novus Biologicals.

### Primary tumor experiments

Liver tumors were cultured as previously described by He et al., [24]. Briefly, they were minced with surgical razor blades and digested at 37°C for one hour in 10 mM HEPES, pH 7.4 and 0.25% w/v collagenase B in Hank’s BSS with Ca^2+^ and Mg^2+^, filtered through a 100 μm cell strainer, preplated and cultured in 20% FCS in DMEM high glucose containing 0.01g/l insulin, 0.01 g/L hydrocortisone hemisuccinate, 1% penicillin-streptamycin, 0.25 mg/L amphotericin B, 1mM phenobarbital and 20 μg/L EGF (Invitrogen or Sigma Aldrich). Cells were not used past passage 8. *In vitro*: In 96-well plates 10,000 cells were plated per well and once 40–50% confluent, Rapamycin and Dasatinib supplied for 24 hours at the indicated concentrations; proliferation was assessed using a BrdU ELISA kit (Roche; performed in quadruplicate three times). *In vivo*: 2.5 x 10^6^ cells in 150 μL Matrigel/PBS were injected bilaterally and subcutaneously into the back flanks of 8–12 week old male wild-type mice. Tumor growth was monitored 3 times per week by measurement with calipers. For treatment, tumors were allowed to either reach approximately 50 mm^3^ or 500 mm^3^ and the mice then treated daily by oral gavage: Rapamycin 7.5 mg/kg and Dasatinib 30 mg/kg (Selleckchem) diluted in 5.1% polyethylene glycol and 5.1% Tween-80.

### Statistical analysis

All values are reported as mean ± standard error of the mean. Unless otherwise specified in the figure legend, statistical significance was determined using a one-way ANOVA for experiments with two or more groups, and Bonferroni post-testing, with p-values <0.05, 0.01, 0.001 and 0.0001 considered to be significant and represented with (*), (**), (***) and (****), respectively. The students t-test was employed for statistical comparison between two groups (Graphpad Prism).

## Results

### APN KO mice have larger HCCs

**Fig 1:**
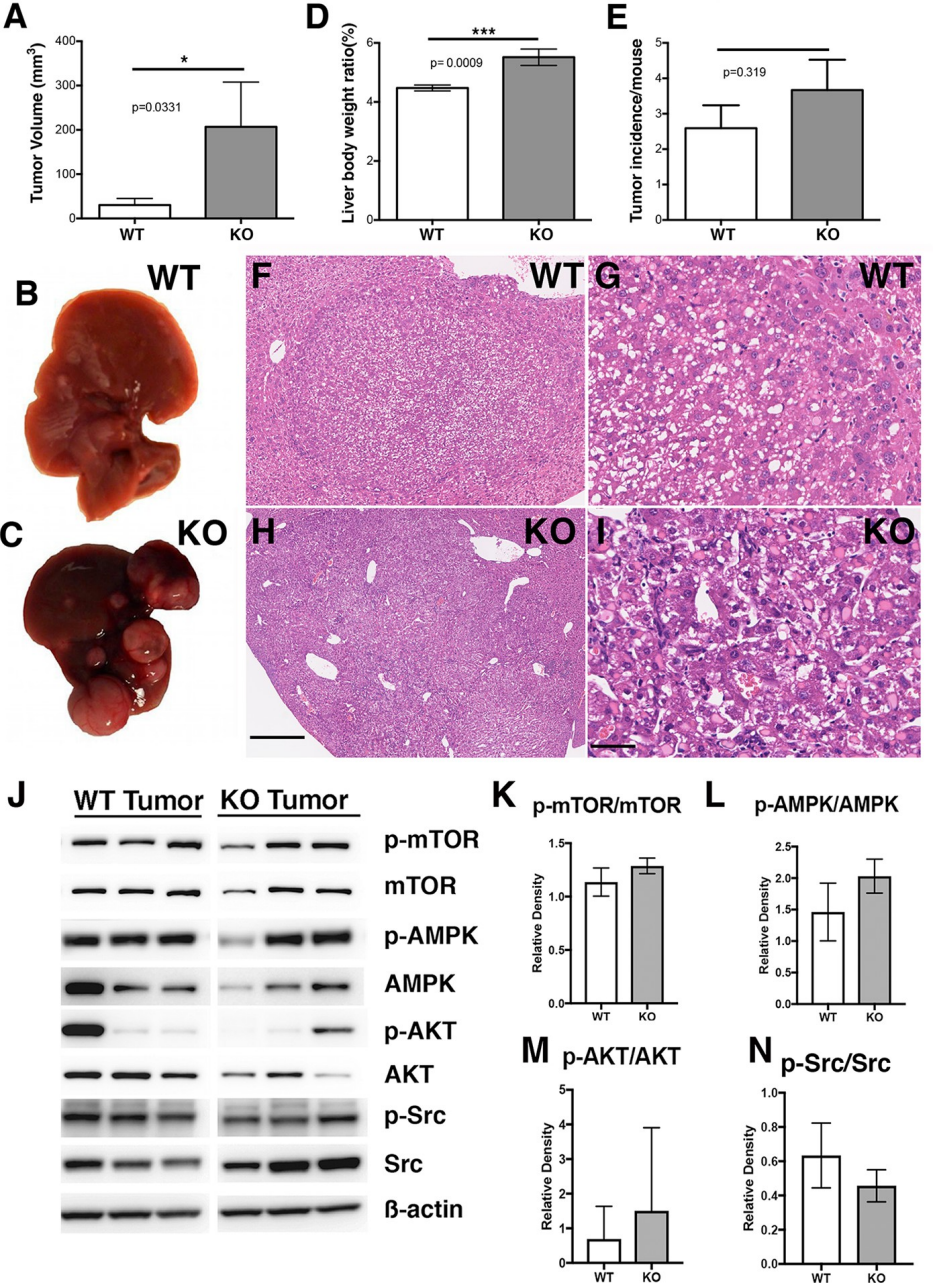
APN KO mice have larger HCCs. Adiponectin KO male mice 9 months post-DEN have **A**, liver tumors 7 times larger than wild type male mice. **B-C**, Representative images of WT and APN KO livers 9 months after DEN treatment. **D,** APN KO mice have increased liver body weight ratio. **E**, Similar tumor incidence between WT and APN KO mice. **F-I**, Representative histology from WT (**F, G**) and APN KO (**H, I**) tumors (scale bars 500 and 50 μM). **J-N**, Western blot and densitometry analyses of WT and APN KO tumors show unaltered mTOR, AMPK, AKT and Src signaling.

HCC was induced in APN KO mice and after 9 months the mice had significantly larger HCCs. The tumors were 6.8-fold larger and total tumor volume was 207 ± 101 mm^3^ versus 30.2 ± 15.0 mm^3^ in the KO mice versus WT controls (p = 0.0331; **Fig 1A–1C**). Liver to body weight ratios increased in APN KO (APN KO: 5.51 ± 0.277%, n = 21 versus WT: 4.47 ± 0.101%, n = 22; p = 0.0009, **Fig 1D**), and between groups there was no difference in tumor incidence (**Fig 1E**). Analyses revealed in WT, 12 pre-neoplastic foci and 23 liver carcinomas, and in the APN KO, 14 pre-neoplastic lesions and 32 carcinomas. Histology analyses illustrated similar pathology in non-tumor regions, and no remarkable change was noted in pathology between WT and APN KO tumors. Inclusion bodies were observed in the tumors of both genotypes and examples of neoplastic pathology at low and high-power magnification are illustrated (**Fig 1F–1I**). No lung metastases were recorded in either genotype and Ki67 staining was increased in APN KO versus WT tumors by 1.4-fold (p = 0.0044; **S1A–S1C Fig**). There was no apparent collagen deposition in either genotype (**S1D and S1E Fig**) and no significant change in metabolic and inflammatory markers (**S2A–S2J Fig**). These data suggest that the absence of APN promotes development of larger and more aggressive liver cancers.

### Establishment of APN KO primary tumor cell lines

APN can modulate HCC tumor cell behavior through specific signaling pathways [25]. Hence, to determine any pro-proliferative signals we undertook western blots and densitometry of phosphorylated and total signaling proteins. And we saw unaltered protein expression of p-mTOR, p-AMPK, p-AKT, p-Src and p-JNK between genotypes in the tumors (**Fig 1J–1N; and S2K and S2L Fig**, **S3 Fig**).

**Fig 2:**
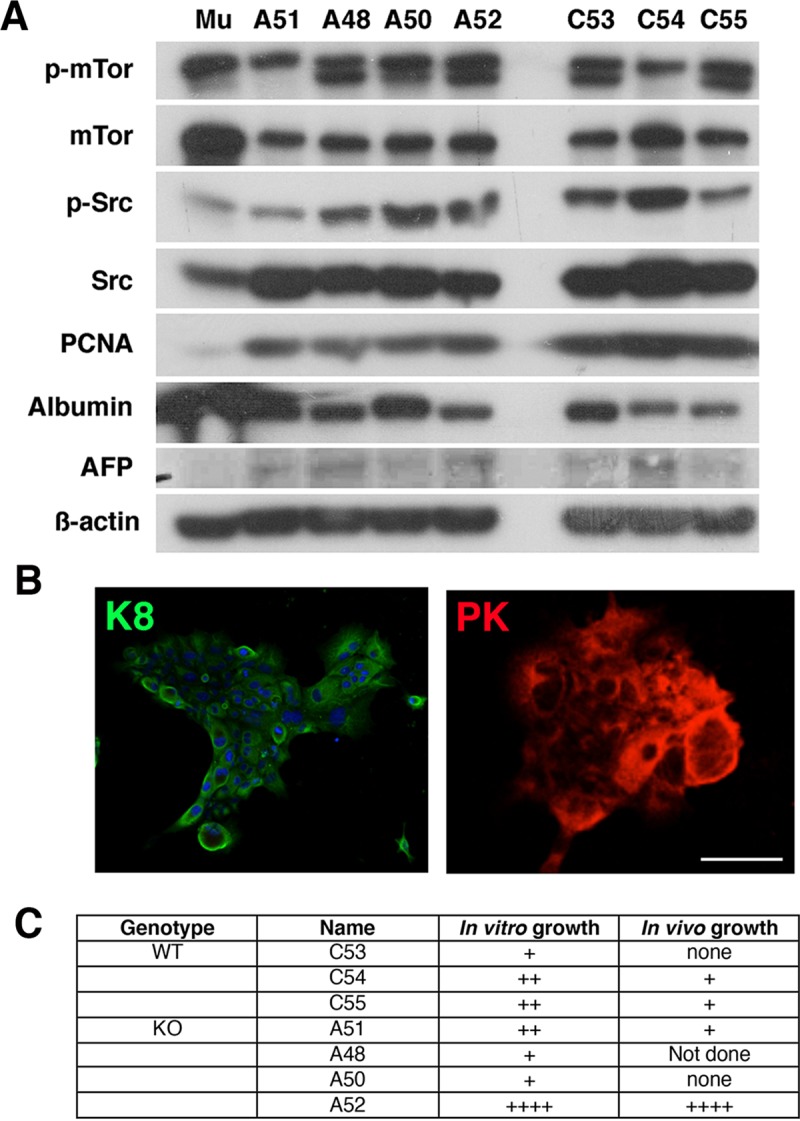
Primary cell characterization. As per the materials and methods, tumors were isolated from mice and cultured. **A**, Primary murine hepatocytes were used as controls (Mu). Western blot revealed the primary cultures of APN KO A51, A48, A50 and A52 and WT C53, C54 and C55 cells to express p-mTOR, mTOR, p-Src, Src, PCNA, albumin and AFP. **B**, Immunofluorescence for K8 and pan-cytokeratin (PK) confirmed that A52 cells were epithelial (Scale bar 20 μM). **C**, Table showing *in vitro* and *in vivo* growth characteristics of WT and APN KO primary cultures. Scale of growth (+ to ++++).

**Fig 3:**
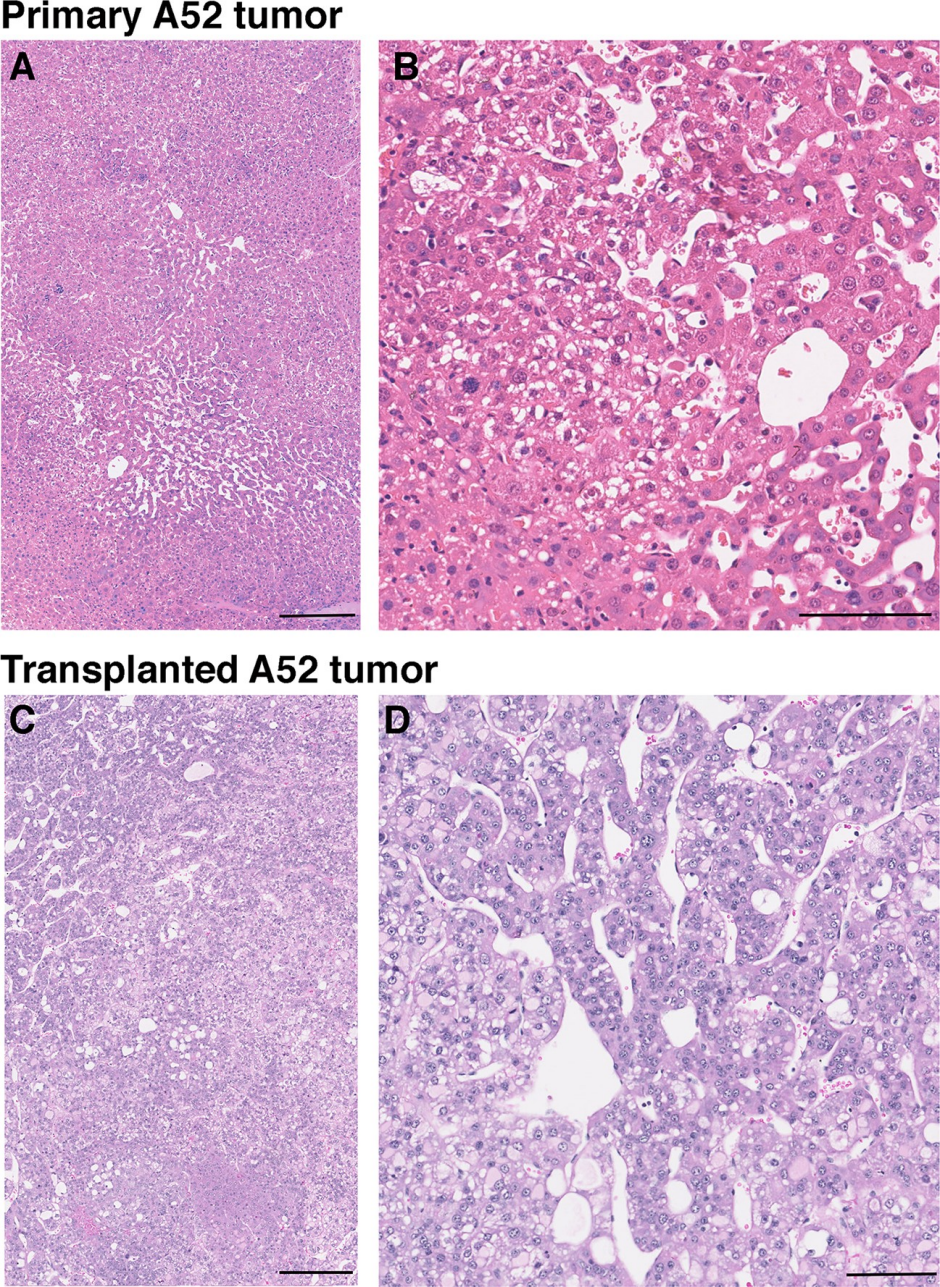
Similar pathology between parental and transplanted A52 tumors. H& E sections of **A and B**, The original APN KO A52 tumor, and **C and D**, Transplanted A52 tumor. Scale bars: A and C 250 μm; and B and D, 100 μm.

In the HCC field there are limited syngeneic cellular models of murine HCC that can be propagated *in vitro* and *in vivo*. We therefore generated primary cultures of WT and APN KO tumor cells and developed a transplantable model system to trial therapeutic strategies. Western blot of the primary culture cell lysates revealed expression of p-mTOR, mTOR, p-Src, Src, PCNA, albumin and α-fetal protein (AFP) (**Fig 2A; S4 Fig**) in WT and APN KO cells. Their epithelial nature was confirmed by immunofluorescence for Keratin 8 (K8) and Pancytokeratin (PK; A52 cultures; **Fig 2B**). Evaluation of growth revealed that WT tumor cells grew poorly *in vitro* and *in vivo* compared to APN KO tumor cells, and only A52 cells had robust growth *in vitro* and *in vivo* (**Fig 2C**). Significantly, the examination of the pathology of the primary and transplanted A52 cells revealed similar morphological similarities (**Fig 3**). On this basis, we chose to use A52 cells for our remaining studies.

### Combinatorial treatment with Rapamycin and Dasatinib restricts HCC growth

**Fig 4:**
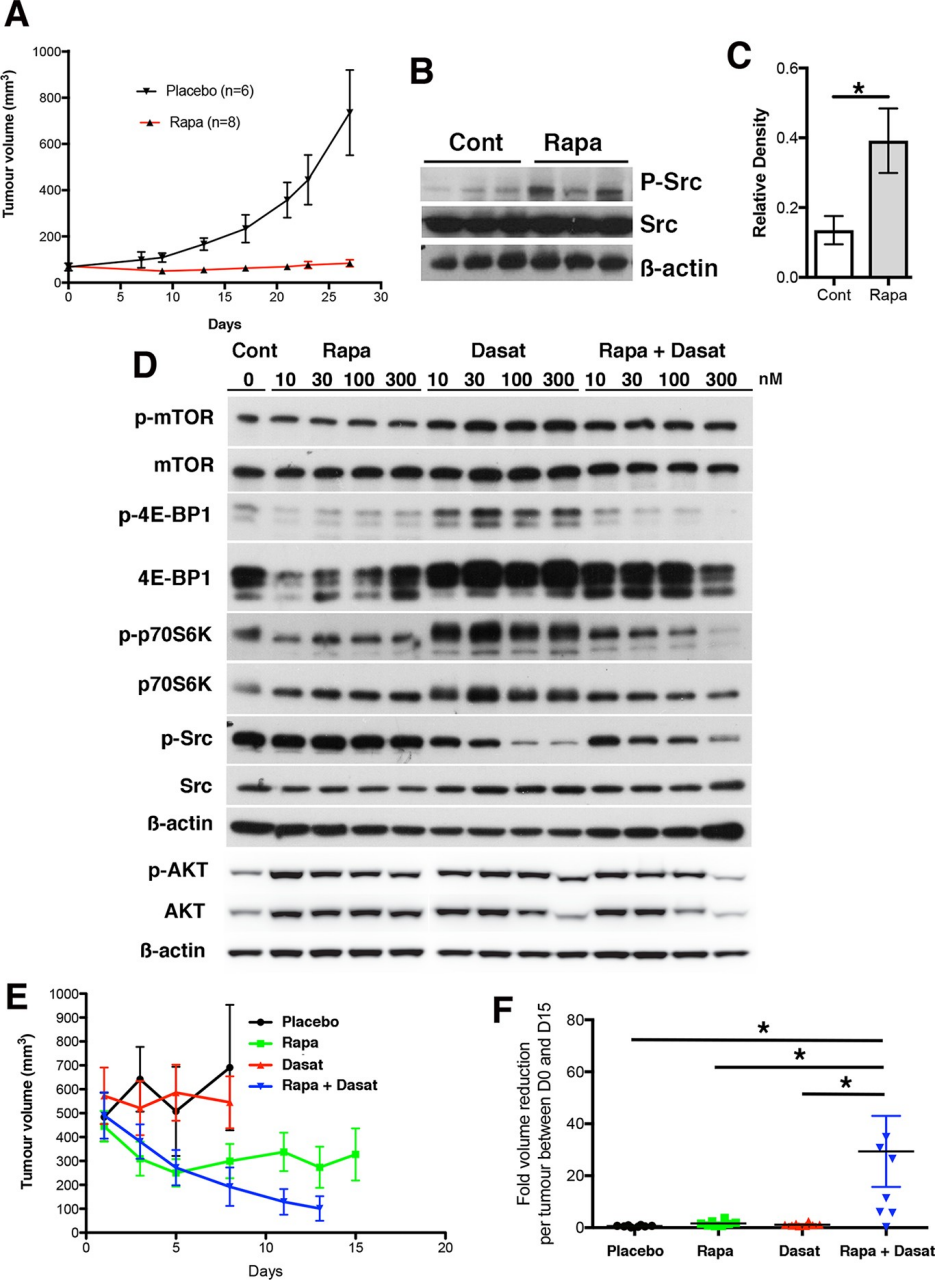
Limiting mTOR and Src activity restricts HCC growth. **A,** A52 subcutaneous tumor growth curves when treated with Rapamycin (Rapa; n = 8) and versus placebo (n = 6). Linear regression demonstrated that Rapamycin (p <0.0001) significantly reduced tumour growth. **B-C**, Western blotting showed a significant increase in p-Src/Src in the Rapamycin treated group (*p<0.05). **D**, Dose-dependent signaling analyses showed that Rapamycin (0–300 nM) reduced 4E-BP1 and p70S6K phosphorylation and Dasatinib (0–300 nM) reduced Src phosphorylation. The combination of Rapamycin and Dasatinib reduced 4E-BP1, p70S6K, Src and AKT phosphorylation. ß-actin loading control. **E**, Growth curves of 500 mm^3^ subcutaneous tumors treated with Dasatinib, Rapamycin, or Rapamycin + Dasatinib (n = 8, all groups). Linear regression analyses showed that Dasatinib or Rapamycin had no effect on tumor growth kinetics. The combination of Rapamycin + Dasatinib reduced tumor growth kinetics (p = 0.0012). **F**, Fold change in tumor volume per tumor for the treatment period Day 0–15 was determined by one-way ANOVA and Bonferroni’s multiple comparisons test. Versus placebo, Dasatinib or Rapamycin had no effect, and Rapamycin + Dasatinib significantly increased the fold-reduction in tumor volume (p = 0.029).

Given that the APN KO and WT HCCs and primary cultures express abundant phosphorylated mTOR and Src we chose to target these pathways. To test the mTOR pathway we treated A52 tumors with the mTOR inhibitor Rapamycin and it effectively restricted tumour growth (p = 0.0001). In discerning signaling differences, we observed increased Src phosphorylation in the Rapamycin treated tumor cohort (p<0.05) (**Fig 4A–4C**). Previous data has shown that mTOR inhibitors in conjunction with Src inhibitors are more efficacious in treating various cancer types, we therefore considered whether this mechanism was functional in A52 cells [9, 26]. To confirm the action of each drug on mTOR and Src phosphorylation, we treated A52 cells with increasing concentrations of Rapamycin or Dasatinib and performed western blots for phosphorylated and total mTOR, 4E-BP1, p70S6K, Src and AKT. Compared to control cells, Rapamycin treatment alone had no effect on p-mTOR, p-Src and p-AKT, but reduced p-4E-BP1 and p-p70S6K levels. Dasatinib treatment did not decrease p-mTOR, p-4E-BP1, p-p70S6K and AKT levels, but did reduce p-Src levels. The combination of Rapamycin and Dasatinib had no effect on p-mTOR, but greatly impeded the phosphorylation of 4E-BP1, p70S6K, Src and AKT (**Fig 4D; S5 Fig**).

Given that the application of both Rapamycin and Dasatinib had a greater inhibitory effect on mTOR and Src activity than either alone, we tested their ability to restrict A52 HCC growth *in vivo*. To model HCC growth as in patients, the tumors were allowed to reach 500 mm^3^ and then treated with each drug alone, or in combination. Analyses of tumor growth rates by linear regression revealed that compared to the placebo group, Dasatinib had no effect on tumor growth and Rapamycin reduced tumor volume, but this was not significant. In contrast, the combination of Rapamycin and Dasatinib significantly decreased tumor growth (p = 0.0012; **Fig 4E**) and reduced the fold volume change per tumor by 29-fold (p = 0.029; **Fig 4F**), and this was significantly different when compared to singular Rapamycin (p = 0.037) and Dasatinib (p = 0.033) treatments.

**Fig 5:**
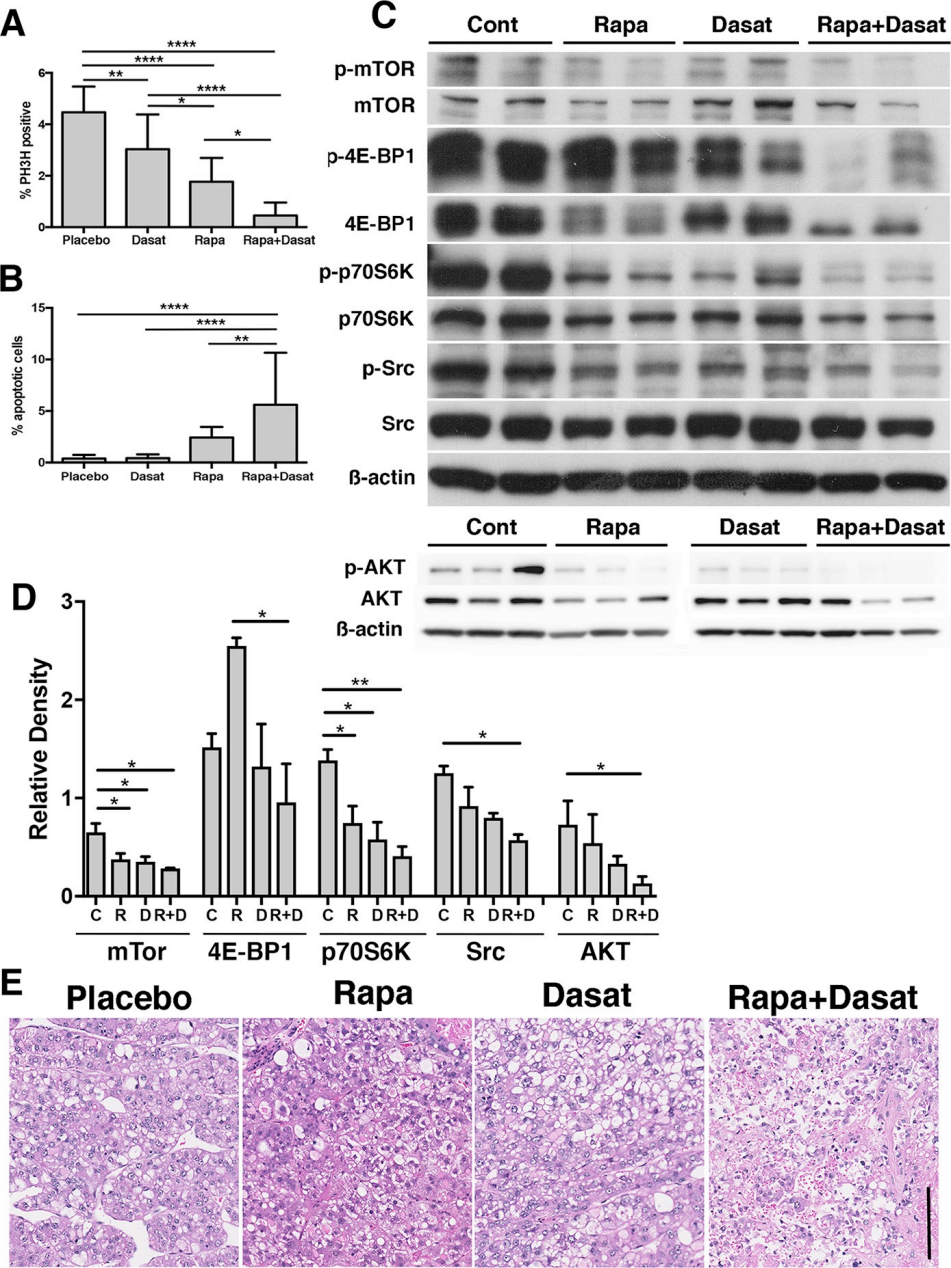
Analyses of tissues after drug treatment. **A**, Histology for phospho-histone H3 (P3H3; n = 4–6 for each group) showed that the Dasatinib (Dasat), Rapamycin (Rapa) and Rapamycin + Dasatinib treatments successively reduced tumor cell proliferation and increased apoptosis. Proliferation: control versus Dasatinib (p = 0.01); Placebo versus Rapamycin (p = 0.0001); control versus Rapamycin + Dasatinib, (p = 0.0001); Dasatinib versus Rapamycin (p = 0.05); Dasatinib versus Rapamycin + Dasatinib (p = 0.0001) and Rapamycin versus Rapamycin + Dasatinib (p = 0.05). **B**, Histology by TUNEL (n = 4–6 for each group) showed that compared to control, only Rapamycin + Dasatinib treatment increased tumor cell apoptosis (p = 0.0001), was significantly greater than that observed for Dasatinib (p = 0.0001) and Rapamycin (p = 0.01) treatments. **C-D,** Representative western blot densitometry analyses showing that compared to control, a small but significant reduction in p-mTOR/mTOR density in all groups (p = 0.05); an increase in p-4E-BP1/4E-BP1 for Rapamycin (p = 0.05) and no significant change in Dasatinib or Rapamycin + Dasatinib; reduced p-p70S6K/p70S6K density for all groups (p = 0.05 for Rapamycin or Dasatinib, and p = 0.01 for Rapamycin + Dasatinib); and reduced p-Src/Src and p-AKT/AKT for control versus Rapamycin + Dasatinib only (p = 0.05). **E**, Tumor pathology was necrotic in the Rapamycin + Dasatinib treatment group.

The dramatic decrease in tumor load we observed could be accounted for in part by a reduction in tumor cell proliferation and an increase in apoptosis, as determined by quantitative immunofluorescence of phosphorylated histone H3 (PH3H) and TUNEL, respectively (**Fig 5A and 5B; S6 Fig**). Compared to placebo, Dasatinib reduced tumor cell proliferation 1.5-fold (p < 0.01) and Rapamycin 2.5-fold (p < 0.0001), while Dasatinib or Rapamycin did not induce tumor cell apoptosis. In contrast, the combination of Rapamycin and Dasatinib reduced HCC proliferation 9.8-fold and increased apoptosis 14.7-fold (p<0.0001 for both). Unexpectedly, in the tumors, unlike cells in culture, mTOR phosphorylation was better inhibited (p<0.05; for all) and 4E-BP1 phosphorylation was only inhibited in the co-treatment group. p70S6K phosphorylation was limited by all three therapeutic approaches (p<0.05 for Rapamycin or Dasatinib, and p<0.01 for Rapamycin plus Dasatinib). Src and AKT phosphorylation was reduced only in the combinatorial cohort (p < 0.05, **Fig 5C and 5D; S7 Fig**). Given these data, we examined tumor pathology and observed that placebo, Rapamycin and Dasatinib groups had aggressive HCC pathology while the combinatorial treated tumors were necrotic (**Fig 5E**). These data suggest that the effects of Rapamycin and Dasatinib restricts mTOR and Src signaling leading to reduced A52 tumor growth.

### Human translational potential

**Fig 6:**
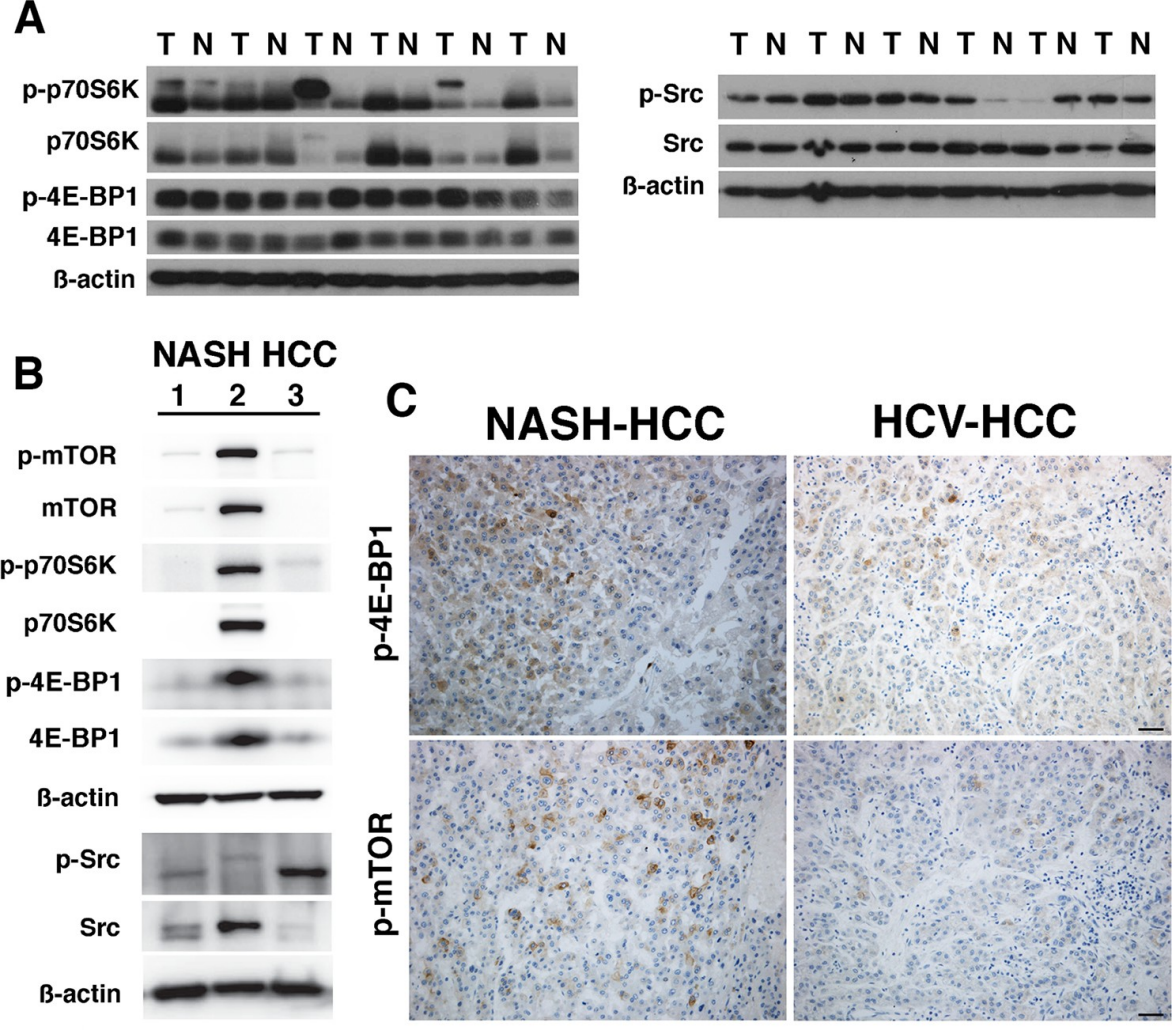
The mTOR and Src pathways are expressed in human HCC. **A**, Western blots of human hepatic non-tumor and tumor from HBV and HCV HCCs illustrate expression of p-p70S6K, p-4E-BP1 and p-Src; and **B**, in human NASH HCCs. **C**, Representative histology of human NASH HCC and HCV-HCC illustrating specific staining for p-mTOR and p-4E-BP1.

To determine if the signaling pathways observed in our animal model are present in human HCC we investigated the expression of the mTOR and Src pathways in human tumor tissues (Westmead Liver Clinic). In six primary pairs of HBV (n = 3) and HCV (n = 3) tumor and non-tumor tissues we observed in the tumors the expression of p-p70S6K (4 from 6), p-4E-BP1 (6 from 6), and p-Src (5 from 6) (**Fig 6A**). In NASH HCC tumors the mTOR pathway was present in one sample and p-Src in 2 (**Fig 6B**, **S8 Fig**). To extend our studies, 10 HCV and 10 NASH HCC samples (Leuven Histopathology Tumor Bank) were examined histologically for p-4E-BP1 and p-mTOR expression. For HCV HCC, 5 from 10 expressed p-mTOR and 6 from 10 were p-4E-BP1 positive, and for the NASH HCC group, 9 from 10 expressed p-mTOR and 8 from 10 were p-4E-BP1 positive (**Fig 6C**). These data suggest that active mTOR and Src signaling is a feature of some human HCCs and their combinatorial targeting may hold promise as a mechanism-based therapeutic approach to treat HCC.

## Discussion

Due to late detection, limited resection options and therapeutics, liver cancer is a poorly treated cancer. Moreover, it is expected that the already high and increasing worldwide rates of overweightness and obesity will translate into increased HCC incidence [27]. Furthermore, this is compounded as the search for new therapeutics is restricted by the limitation of transplantable models that resemble human disease and the use of genetic models that are unacceptably long and expensive. To address this, we used APN KO mice and developed a transplantable syngeneic model and validated its potential using known inhibitors, and then performed a comparative signaling analyses of human HCC. Significantly, we find that A52 tumors when treated with the combination of Rapamycin and Dasatinib are dramatically reduced in size and we show that these pathways are present in a subset of human HCC driven by HBV, HCV and NASH.

mTOR expression has been extensively studied in HCC, but its inhibition by small molecules has been shown to be an ineffective way to treat HCC. By example, trials with the mTOR inhibitor Everolimus alone or when combined with sorafenib offered no survival advantage compared to placebo in patients with advanced HCC [12, 14]. In tumors of the colon, prostate and breast, Src is highly expressed and is associated with aggressive migratory cell behavior, differentiation and recently metabolism [28–30]. In HCC, Src is expressed in approximately 50% of human tumors derived from either HBV or HCV [31], but few studies have explored Src’s role in other molecular pathways such as mTOR or the utility of specific Src inhibitors to restrict HCC growth.

A major reason for the failure of mTOR targeted therapies is feedback activation of the strong and pro-survival AKT and the proto-oncoprotein Src. Emerging data shows that non-RTKs like Src and other Src-kinases can support PI3K/AKT/mTOR signaling by modulating cell-cycle progression and survival [32, 33], and in turn mediate RTK signaling that activates mTOR [34]. Additionally, Src can interact and activate AKT [35], and in other models independent of AKT, activate the mTORC1 complex [36], and separately modulate p70S6K activity [37]. Thus, there are potentially multiple levels for crosstalk between the Src and mTOR pathways. Studies in breast cancer and human non-small cell lung cancer (NSCLC) have shown that Rapamycin maintains AKT and Src activity. The combination of Rapamycin and Dasatinib induced tumor regression in a genetic mouse mammary tumor virus-polyomer middle T antigen model [9], and in NSCLC suppressed Src and AKT phosphorylation [26]. In line with our expectations, we observed that Rapamycin failed to reduce tumor growth and there was no effect on AKT and Src activity. Similarly, Src’s action *in vivo* was akin to that of the placebo and there was no effect on AKT phosphorylation. In direct contrast, the use of both inhibitors *in vivo* further reduced mTOR and p70S6K phosphorylation and strikingly reduced Src and AKT phosphorylation. Importantly, the drug combination altered tumor pathology, increased necrosis and reduced tumor cell proliferation, and resulted in cancers that were 29-fold smaller than placebo and had regression of the aggressive phenotype. This study is the first to show in a pre-clinical HCC model the efficacy of using both Rapamycin and Dasatinib to treat and restrict HCC growth.

To support our data, we examined mTOR and Src pathway activity in a small cohort of tissue samples from HBV, HCV and NASH HCC. In frozen tissues of HBV and HCV HCC both pathways are expressed, and in the three NASH HCCs the mTOR pathway is less active, but this could just be a function of the time of resection to being snap frozen, while histological the mTOR pathway was expressed, and is in agreement with a previous study examining p-mTOR and p-4E-BP1 expression in NASH HCC [38]. Previous studies have histologically shown the expression of Src and p-Src in HCC types [31]. We attempted similar studies with three commercial antibodies against p-Src and this did not parallel Src staining to similar cellular structures. Thus, it remains to be determined how active Src can be evaluated in the clinical setting from tumor biopsies. Therefore, we suggest that further studies of human HCC mTOR and Src expression are required, prior to trialing a combinatorial strategy.

Our data also shed caution on choosing the correct experimental design in validating HCC inhibitors. While *in vitro* the combined action of Rapamycin and Dasatinib on A52 proliferation was no better than that observed with Rapamycin alone, and yet *in vivo* the combinatorial approach was very effective and suggests that it may limit other tumor functions. Additionally, in preliminary experiments where Rapamycin treatment was given to mice with small A52 tumors, the inhibition of tumor growth was robust, but in larger established tumors of 500 mm^3^, Rapamycin had an initial response, but overtime the tumors increased resistance. These data suggest, that perhaps when considering HCC therapeutic experiments in mice, larger syngeneic tumors should be used, to better reflect established immune cell, vascular and stromal effectors, as seen in HCC patients.

Due to the high and growing rates of obesity and of the different cancer types, HCC demonstrates perhaps the greatest increase in risk [39]. Significantly, serum APN levels are directly and inversely associated with an increasing adipocyte mass and is a key player in the pathogenesis of type 2 diabetes, metabolic syndrome and NASH. Studies have shown that low serum adiponectin is associated with progression to chronic liver disease [40, 41]. Given that previous studies have observed APN KO mice to develop HCC after chemical insults [20, 21, 42], we chose to use the liver carcinogen DEN to more accurately gauge the role of APN in HCC growth. As expected, all WT and KO mice generated liver tumors without fibrosis. This could reflect emerging data demonstrating that up to 50% of NASH HCC develops in the absence of advanced fibrosis and inflammation [43–45]. Interestingly, while no difference was observed in the number of tumors, there was marked anisokaryosis, earlier appearance, increased Ki67 staining and greater tumor volumes, indicating that the APN KO HCCs are more aggressive than WT. Moreover, due to numerous metabolic and tumor studies, it was our expectation that APN absence would coincide with active AMPK/mTOR signaling in APN KO HCCs [16]. Published data concerning the manner through which APN inhibits HCC growth is divergent, probably due to the utilization of human HCC cell lines and nude mouse models. By example, Saxena et al., inoculated mice with HepG2 cancer cells and in this context, over-expression of APN impeded HCC growth through JNK and mTOR [25]. In contrast, our analyses revealed unaltered JNK, AKT, Src and AMPK/mTOR pathway activity. Given this data, and that the tumors in the APN KO mice presented with unaltered markers of metabolism and inflammation, it suggests that other mechanisms promote APN KO tumor growth; and these we are now investigating.

To generate primary cultures of HCC cells we utilized similar protocols from other cancer biology fields, and pre-plating to remove fibroblasts [24, 46]. Of the seven tumors dissociated, they all expressed the mTOR and Src pathways, markers of hepatocytes; albumin and AFP; and epithelial markers; keratins. Only A52 cells gave reproducible growth *in vitro* and *in vivo* and the pathology of the original A52 and transplanted tumors was similar. It remains to be determined what genetic events drive A52 tumorgenicity and how further passaging through mice and in culture will alter their tumorigenesis.

Taken together, we have established a new murine model of human HCC in the context of reduced serum APN. Using this model, we illustrate that the combinatorial targeting of mTOR and Src with the approved drugs Rapamycin and Dasatinib restricts growth *in vivo*, and in considering human HCC we find activity of these pathways. Whilst the underlying molecular mechanisms as to how Src and mTOR regulate each other’s function remains to be determined, our data suggests that this targeted combination effectively reduces tumor growth and thus should be explored for the treatment of HCC.

## Supporting information

S1 Fig APN KO tumors have increased tumor cell proliferation and there is no hepatic fibrosis. Representative Ki67 staining of **A**, WT (n = 6 mice) and **B**, APN KO (n = 6 mice) tumor sections, and **C**, percentage (%) of Ki67 positive cells per field (p = 0.0044, by t-test). Examples of Sirius red staining from WT and APN KO livers, illustrating limited hepatic fibrosis in both genotypes. Scale bar 250 μm.
(TIF)

S2 Fig APN KO have unaltered signaling, metabolic, injury and inflammatory markers. Serum evaluations reveled no differences between genotypes of **A**, glucagon; **B**, insulin; **C**, leptin; **D**, IL-6; **E**, TNFα; **F**, AST; **G**, ALT; and by qPCR for the tumor inflammatory markers **H**, IL-6, **I**, TNFα, and **J**, CD68. Active JNK is not associated with APN KO HCC growth. **K and L,** Western blot and densitometry analyses show unaltered p-JNK/JNK protein in WT and APN KO livers and tumors.
(TIF)

S3 Fig Original Western blots for Fig 1. (TIF)

S4 Fig Original Western blots for Fig 2. (TIF)

S5 Fig Original Western blots for Fig 4. (TIF)

S6 Fig Examples of PH3H and TUNEL staining from A52 tumors treated as placebo or with Rapamycin and Dasatinib. Frozen sections were stained for PH3H (**A**) or TUNEL (**B**) (green) and counterstained with DAPI (blue). Images 40x magnification.
(TIF)

S7 Fig Original Western blots for Fig 5. (TIF)

S8 Fig Original Western blots for Fig 6. (TIF)
