# Supplementary figures and images for: Targeting mTOR and Src restricts hepatocellular carcinoma growth in a novel murine liver cancer model
Source: PLoS One. 2019 Feb 22;14(2):e0212860. doi: 10.1371/journal.pone.0212860 (PMC6386388; doi:10.1371/journal.pone.0212860)

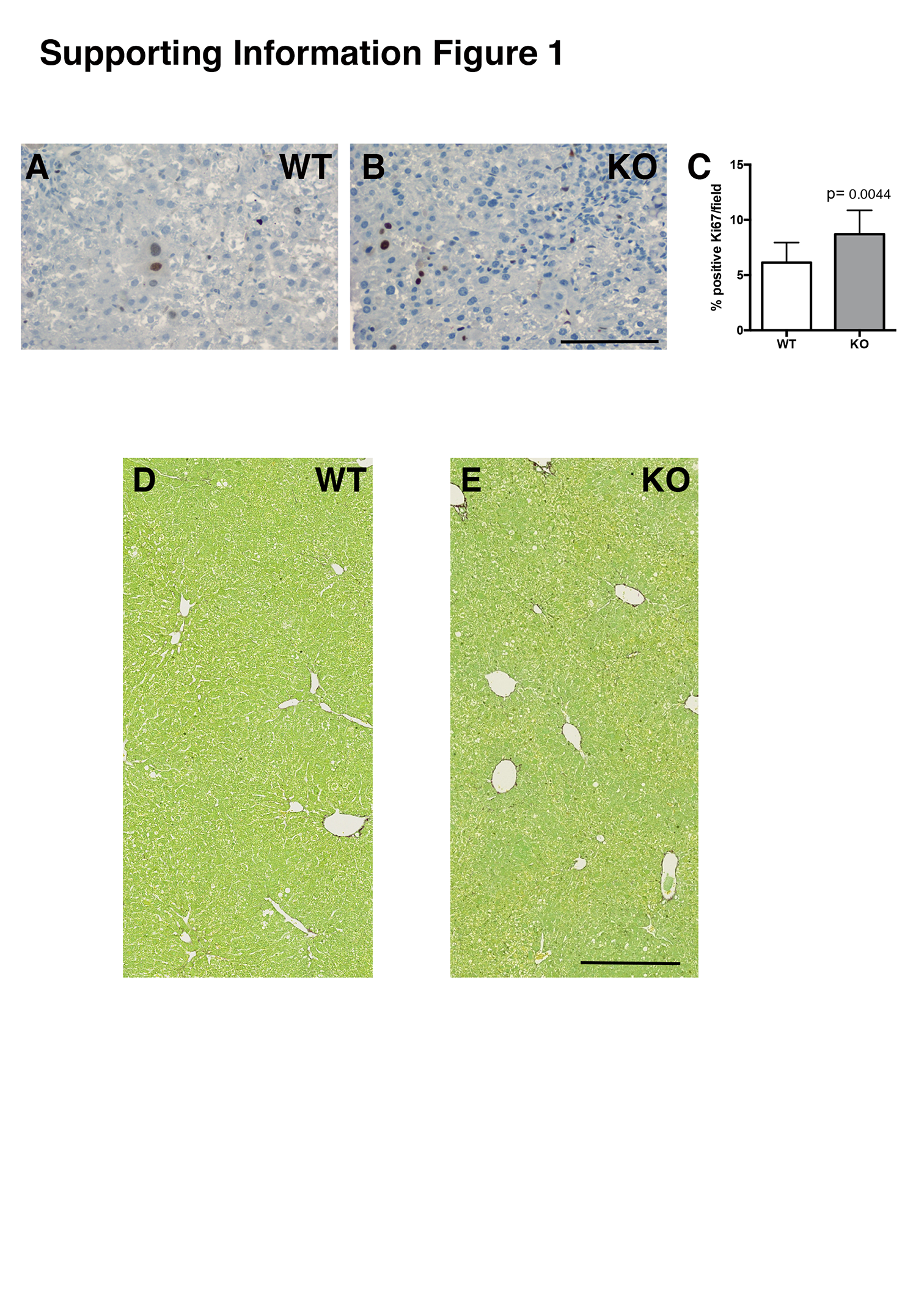

Supplement: S1 Fig — Representative Ki67 staining of A, WT (n = 6 mice) and B, APN KO (n = 6 mice) tumor sections, and C, percentage (%) of Ki67 positive cells per field (p = 0.0044, by t-test). Examples of Sirius red staining from WT and APN KO livers, illustrating limited hepatic fibrosis in both genotypes. Scale bar 250 μm. (TIF) [file pone.0212860.s001.tif]

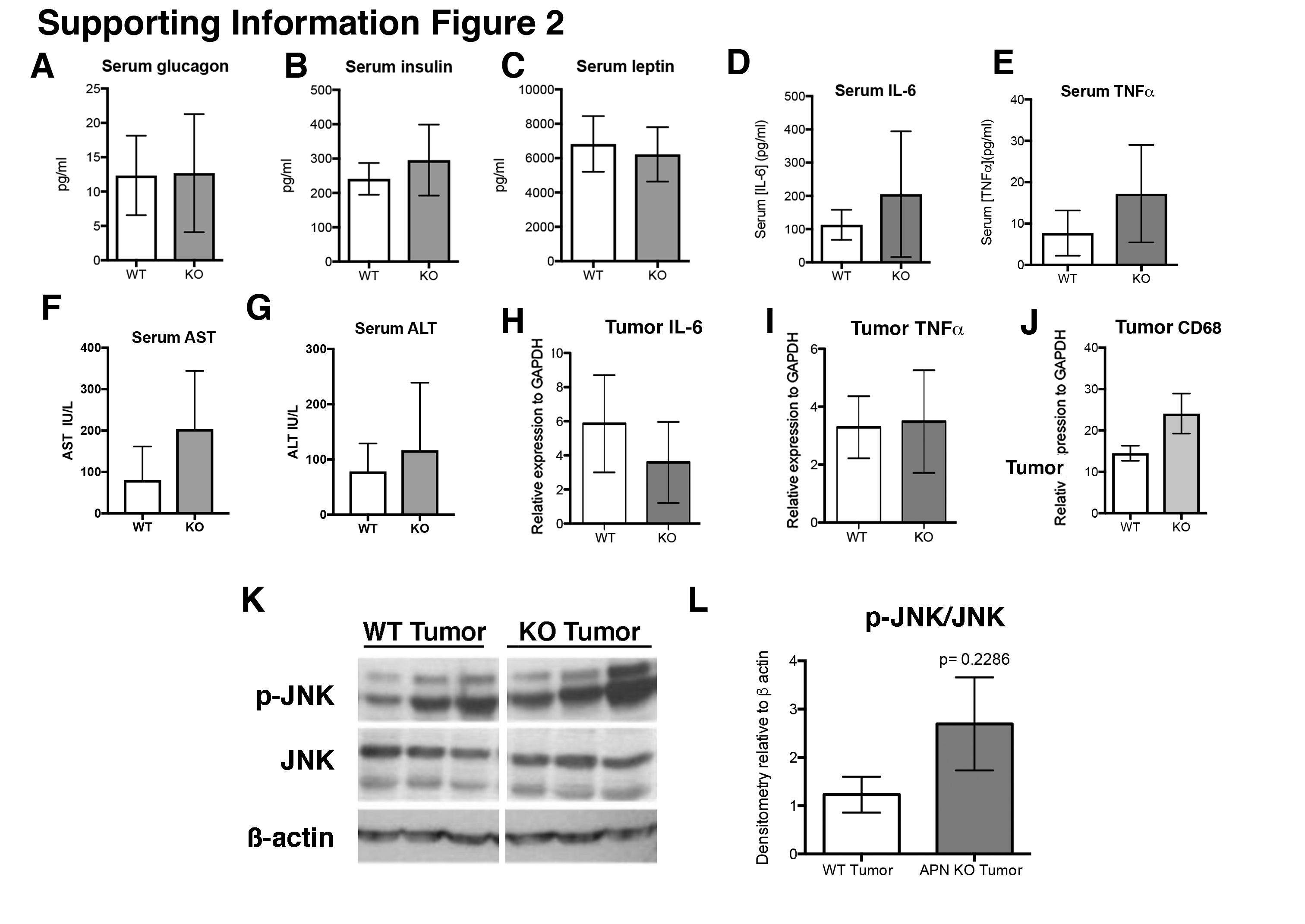

Supplement: S2 Fig — Serum evaluations reveled no differences between genotypes of A, glucagon; B, insulin; C, leptin; D, IL-6; E, TNFα; F, AST; G, ALT; and by qPCR for the tumor inflammatory markers H, IL-6, I, TNFα, and J, CD68. Active JNK is not associated with APN KO HCC growth. K and L, Western blot and densitometry analyses show unaltered p-JNK/JNK protein in WT and APN KO livers and tumors. (TIF) [file pone.0212860.s002.tif]

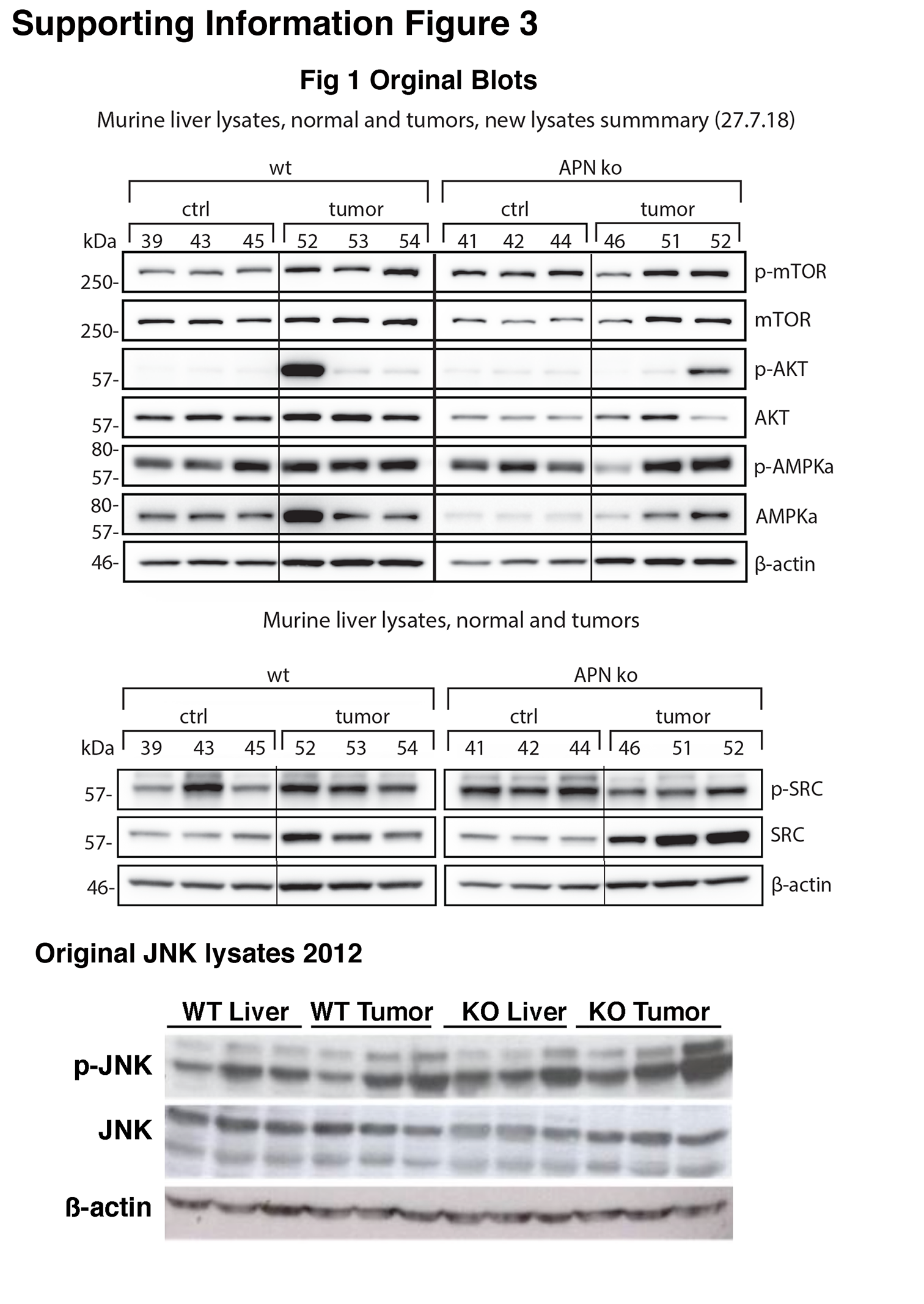

Supplement: S3 Fig — (TIF) [file pone.0212860.s003.tif]

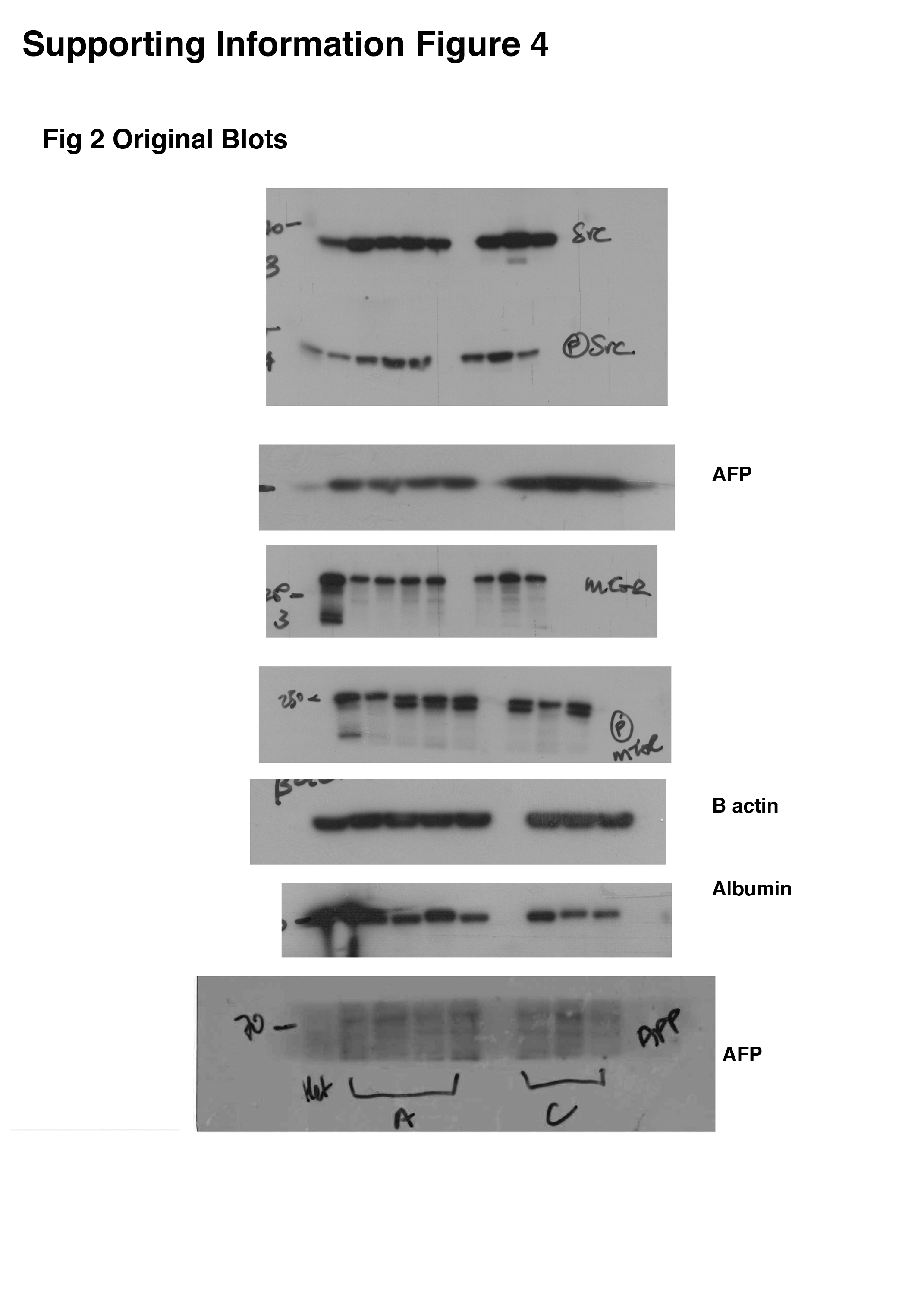

Supplement: S4 Fig — (TIF) [file pone.0212860.s004.tif]

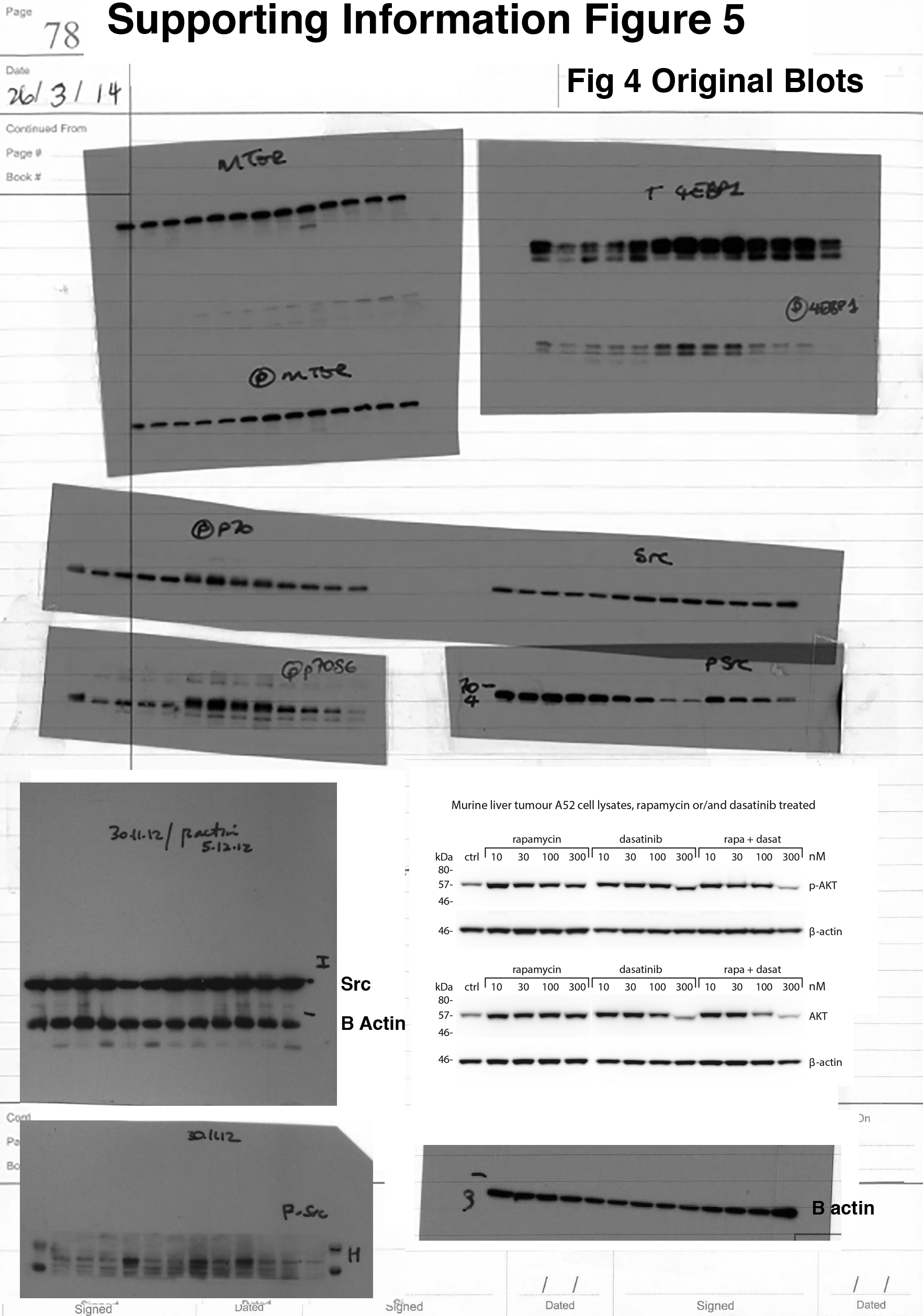

Supplement: S5 Fig — (TIF) [file pone.0212860.s005.tif]

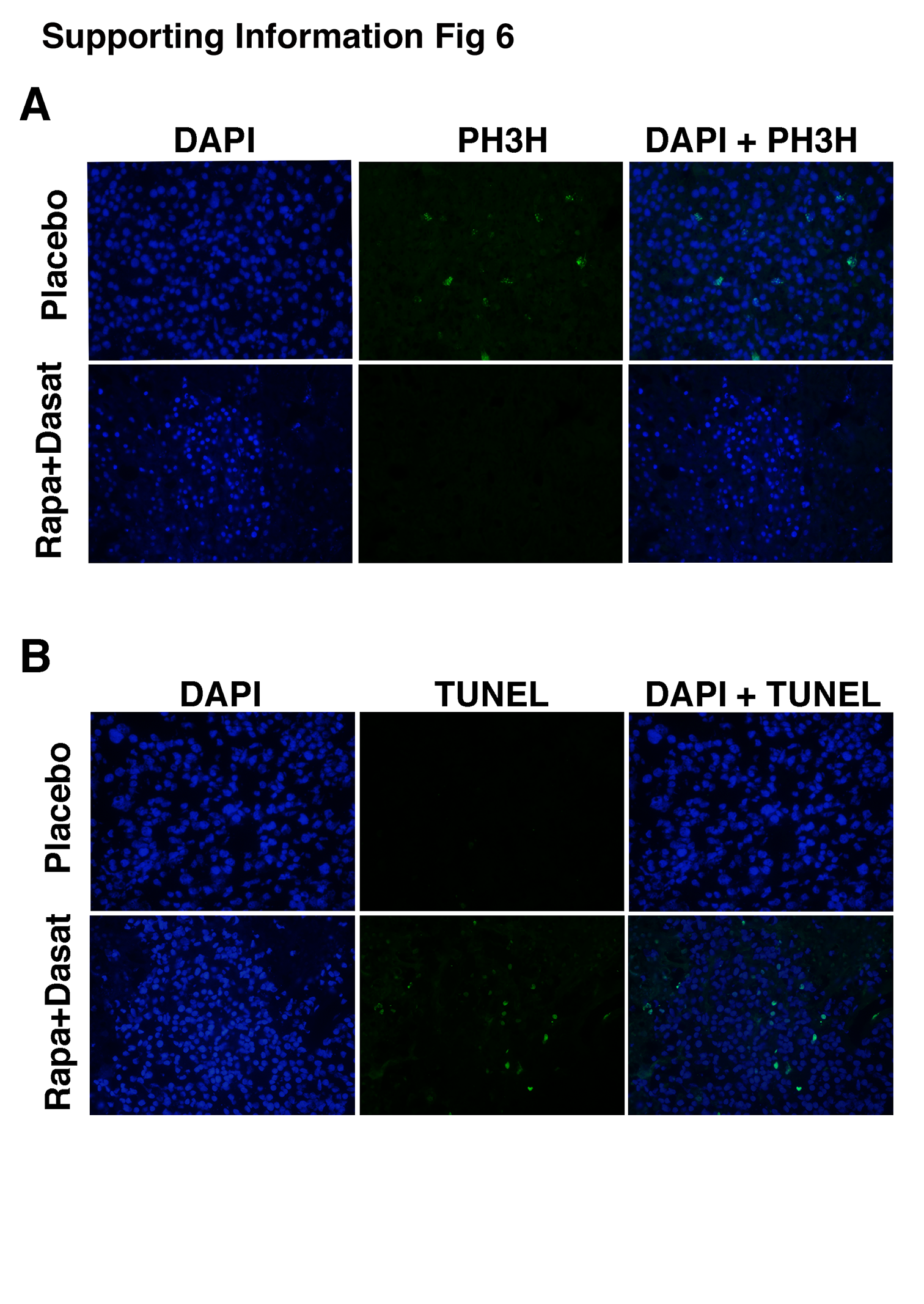

Supplement: S6 Fig — Frozen sections were stained for PH3H (A) or TUNEL (B) (green) and counterstained with DAPI (blue). Images 40x magnification. (TIF) [file pone.0212860.s006.tif]

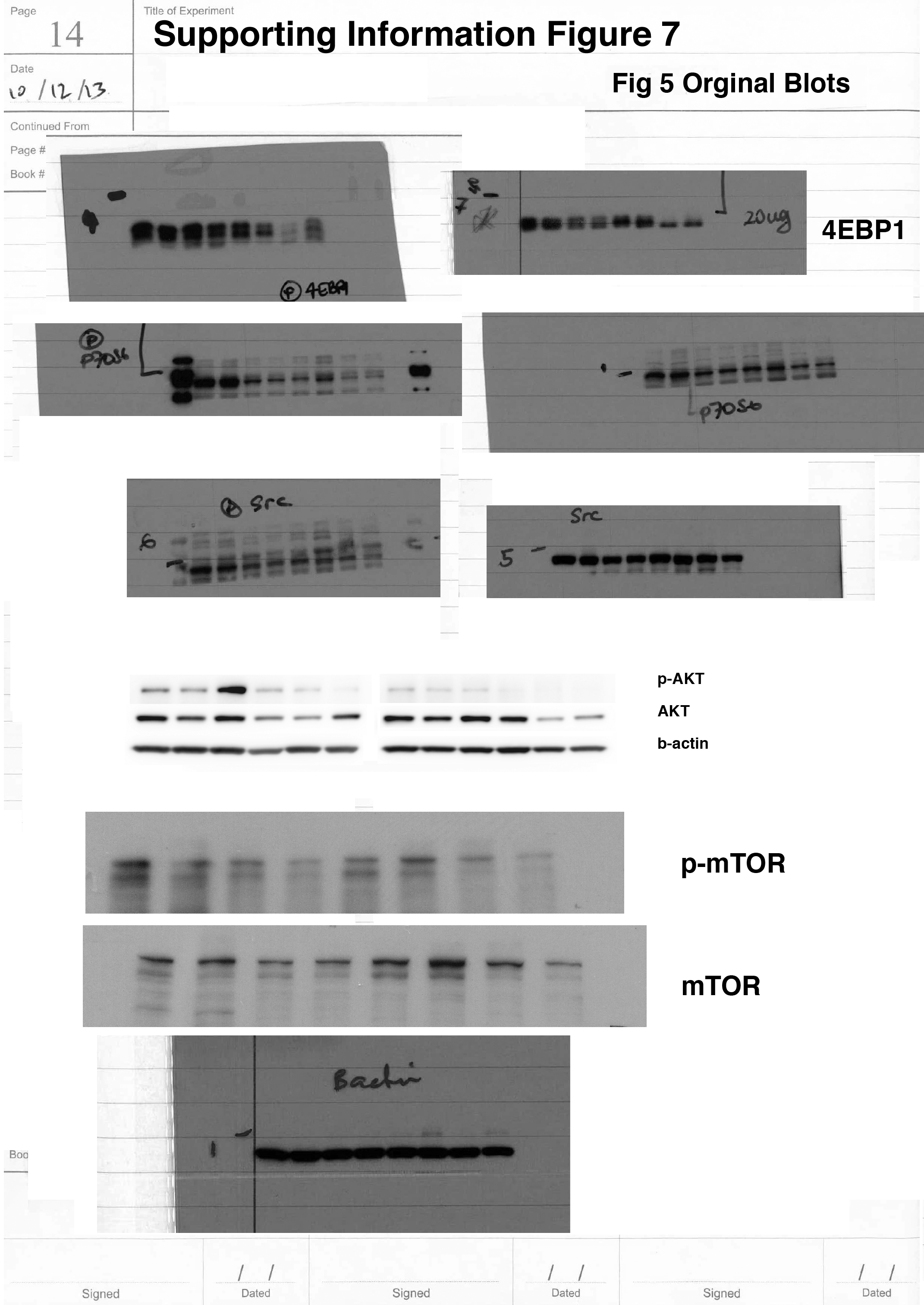

Supplement: S7 Fig — (TIF) [file pone.0212860.s007.tif]

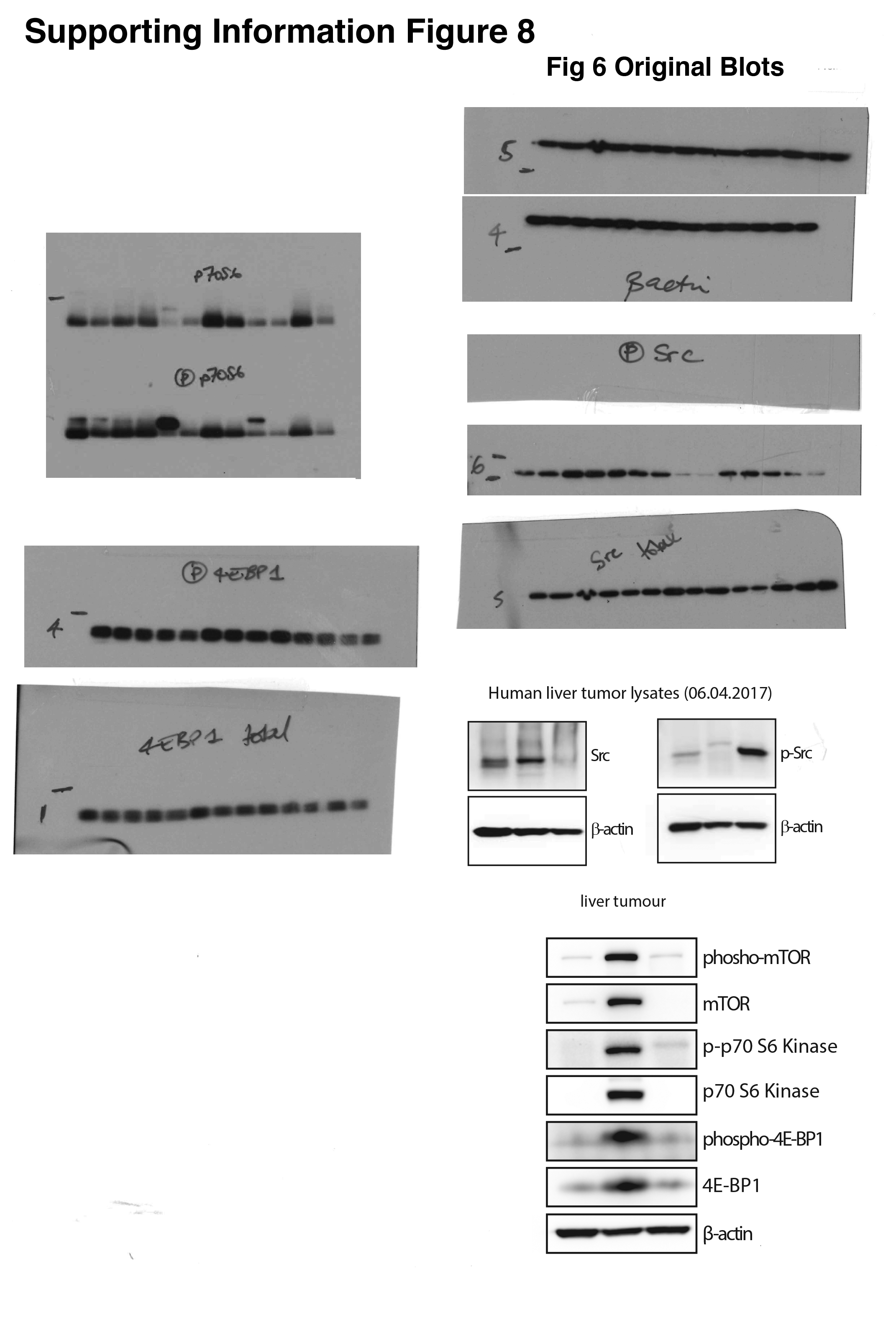

Supplement: S8 Fig — (TIF) [file pone.0212860.s008.tif]
